# Improving the health of workers with a low socioeconomic position: Intervention Mapping as a useful method for adaptation of the Participatory Approach

**DOI:** 10.1186/s12889-020-09028-2

**Published:** 2020-06-19

**Authors:** R. Schaap, F. G. Schaafsma, A. R. Bosma, M. A. Huysmans, C. R. L. Boot, J. R. Anema

**Affiliations:** Amsterdam UMC, Vrije Universiteit Amsterdam, Department of Public and Occupational Health, Amsterdam Public Health research institute, Amsterdam, The Netherlands

**Keywords:** Workers, Low socioeconomic position, Work functioning, Workplace intervention, Intervention Mapping, Participatory Approach, Positive Health approach, Occupational health professional

## Abstract

**Background:**

Workers with a low socioeconomic position (SEP) have a higher risk for health problems and premature dropout from the workforce. Unfavorable working conditions and unhealthy behaviors are more prevalent among this group of workers. The Participatory Approach (PA), is an evidence-based method to identify and solve problems at the workplace related to health issues of the worker. Health problems among workers with a low SEP are usually caused by an interplay of problems in and outside the workplace. To solve health problems on multiple life domains for workers with a low SEP we aim to adapt this approach to a broader perspective.

**Methods:**

An Intervention Mapping (IM) protocol was used to adapt the PA. First, a needs assessment was conducted combining literature with data from interviews and focus groups with workers with a low SEP, employers and occupational health professionals (OHPs). Based on the needs assessment a program goal and performance and change objectives were defined, which resulted in methods and practical strategies to solve problems on multiple life domains. Based on the results of these steps, the PA was adapted and an implementation and evaluation plan were developed.

**Results:**

The needs assessment confirmed that an interplay of problems on multiple life domains affect work functioning and health of workers with a low SEP. Moreover, they perceived difficulties with solving problems or used passive or avoidant coping styles towards these problems. The program goal is to identify and solve problems on multiple life domains that affect healthy functioning at work. To achieve this workers need support from OHPs to solve problems. The PA protocol and materials were adapted using theoretical concepts of the Self-Determination Theory (SDT), which resulted in the Grip on Health intervention. For OHPs a training was developed on how to implement this intervention in practice. The intervention will be evaluated in a pilot implementation study among workers with a low SEP and other relevant stakeholders.

**Conclusions:**

IM was a valuable tool for the adaptation of the PA to better support workers with a low SEP to improve their work functioning and health from a broader perspective.

## Background

Socioeconomic health inequalities are a major societal problem. Workers with a low socioeconomic position (SEP) have a higher risk for health deterioration and premature mortality [1–3]. Therefore, morbidity and mortality rates are generally higher than among workers with a high SEP [4, 5]. Workers with a low SEP may also be more prone to health problems, because unfavorable physical and psychosocial working conditions and unhealthy behaviors are more prevalent among this group of workers [6, 7]. Unfavorable working conditions and unhealthy behaviors are linked to poor health outcomes, which increases the risk for a disability and premature dropout from the labor market [8–10]. Hence, workers with a low SEP are more likely to be unemployed or stop working due to a disability, as compared to workers with a high SEP. Furthermore, dropout from work is likely to lead to further deterioration of health [11]. To prevent work disability among workers with a low SEP it is important to improve work functioning and health of workers with a low SEP which can be achieved by a workplace intervention.

In the past decades there has been a growing awareness for interventions at the workplace that aim to solve health risks at the workplace through involvement of relevant stakeholders. One of these interventions is the Participatory Approach (PA). The effectiveness of the PA has been extensively investigated and these studies have shown that the PA had a positive impact on physical and mental health outcomes and return to work (RTW) [12, 13]. The PA consists of a stepwise process to identify and solve problems at the workplace in a participatory way [14]. This process is guided by an independent occupational health professional (OHP), wherein equivalent and active input of the worker, supervisor and other relevant stakeholders at the workplace is required and together they reach consensus on the most important problems and solutions [15]. Stakeholder involvement may lead to a higher acceptance and implementation of solutions [16, 17]. Moreover, participation of stakeholders may also lead to a better adherence to solutions, which increases the chance that solutions are sustained over time [13]. Gradually the PA has been increasingly implemented in occupational health practice. Herein, the PA originally had an organizational preventive approach and was later on adapted to an individual (RTW) approach [15, 18].

Although the PA is a promising method to reduce health risks at the workplace, this approach solely focuses on problems at the workplace and does not take into account that problems outside the workplace may also interfere with work functioning and health. Workers with a low SEP often face problems on multiple life domains [19], e.g. next to musculoskeletal problems experienced at the workplace, they could also have psychosocial problems or poor housing conditions. According to the new concept of health ‘The Positive Health approach’ the lack of ability to adapt and self-manage physical, emotional and social challenges of life could all be considered as health problems [20]. In this approach health is more than the absence of disease, as one’s health status can be determined by multiple life domains. So, to improve work functioning and health of workers with a low SEP more effectively, the PA might extend its focus to identify and solve problems both in and outside the workplace. Therefore, the aim of this study is to adapt the PA to improve work functioning and health of workers with a low SEP from a broader perspective.

## Methods

**Fig. 1:**
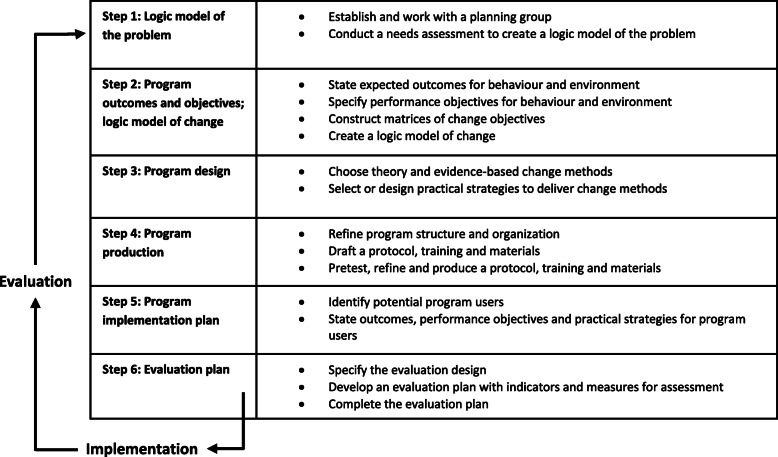
The six steps of Intervention Mapping adapted from Eldredge et al. 2016 [21]

This paper describes the process of adaptation of the PA (Fig. 1), guided by the six steps of an Intervention Mapping (IM) protocol for development, implementation and evaluation of theory and evidence-based health promotion interventions [21]. IM is not rigid, it is an iterative process which makes it possible to move back and forth between steps, and each step is based on previous steps. Moreover, IM stimulates involvement of stakeholders during the entire process to tailor interventions to the needs and wishes of these stakeholders. The Medical Ethics Review Committee of the VU University Medical Center approved the study protocol and confirmed that the Medical Research Involving Human Subjects Act does not apply to this study. All participants signed informed consent before participation.

### Step 1: logic model of the problem

In the first step, a planning group was established for the whole IM process. Next, a needs assessment was conducted which combined evidence from literature with data from six semi-structured interviews with workers with a low SEP working in a steel factory and from two focus groups with OHPs (i.e. 2 occupational health experts, 1 occupational physician, 1 employability coach and 1 occupational social worker) and employers (i.e. 1 health and safety manager and 6 human resource managers). Themes that were discussed in the interviews and focus groups were: 1) the need for discussing problems on multiple life domains, 2) the content of the different steps of the PA, 3) the involvement of relevant stakeholders in and outside the workplace, 4) what type of solutions and in what way solutions can be implemented, 5) the need for a preventive intervention, 6) in what way workers with a low SEP can be reached, and 7) important preconditions for the implementation of the intervention in occupational health practice. In additional file 1, interview guides can be found for the interviews and focus groups. Interviews and focus groups were audio-recorded and transcribed verbatim. Thereafter, the transcripts were summarized and combined with evidence from literature. The needs assessment provided insight into work functioning and health problems of workers with a low SEP and behaviors and underlying determinants that may cause these problems. Furthermore, the needs assessment provided insight into environmental factors and the underlying determinants for these factors that may also cause work functioning and health problems among workers with a low SEP. This resulted in a logic model of the problem. Based on this model a program goal was formulated to improve work functioning and health of workers with a low SEP from a broader perspective.

### Step 2: program outcomes and objectives – logic model of change

In the second step, behavioral and environmental outcomes were developed to achieve the program goal. Behavioral and environmental outcomes were derived from the behaviors and environmental factors that were described in the logic model of the problem. For each of these outcomes, performance objectives were specified, which describe in detail what needs to be done to accomplish the behavioral or environmental outcomes. This resulted in a logic model of change. Thereafter, theoretical concepts were selected to change the performance objectives. Theoretical concepts were based on the behavioral and environmental determinants. Next, matrices of change objectives were constructed; for each behavioral and environmental performance objective strategies linked to theoretical concepts were formulated, to describe what needs to be done to accomplish the performance objectives.

### Step 3 & 4: program design and program production

In the third step, the design of the PA with a broader perspective on health was developed consisting of theory and evidence-based change methods to influence the change objectives for the behavioral and environmental outcomes in step 2. Next, practical strategies were identified to deliver the change methods. In the fourth step, the program structure and organization of the PA with a broader perspective were described in an intervention program, training and materials. All gathered information from the previous steps was synthesized and translated to adapt the PA.

### Step 5 & 6: program implementation and evaluation plan

In the fifth step, a plan for the implementation of the adapted PA was developed. In the implementation plan potential users of the PA were specified. Next, program outcomes, performance objectives and practical strategies were developed for the users to enable optimal delivery. In the sixth and final step of the IM process, an evaluation design was chosen and a plan for the evaluation of the PA was developed to investigate the implementation of the adapted PA in practice.

## Results

### Step 1: logic model of the problem

#### Planning group

The planning group consisted of 3 health scientists (RS, AB, CB), 2 occupational health physicians (FS, JA) and 1 ergonomist (MH). This multidisciplinary planning group was established to adapt the PA for workers with a low SEP. Furthermore, throughout the IM process relevant stakeholders at the workplace were consulted, namely workers with a low SEP, OHPs and employers.

#### Needs assessment

**Health problems among workers with a low SEP**

Literature on the perception of health among workers with a low SEP showed that health has been described as a multidimensional concept [22, 23]. This is in line with the ‘Positive Health approach’, which defines health as the ability to adapt and self-manage, in the light of physical, emotional and social challenges of life [20]. In this approach health is a dynamic phenomenon that should be seen as an integral part of life, rather than something that is only considered when illness occurs. Research shows that this concept is highly appreciated, as it addresses people as more than just their illness, and people themselves can decide what is important to them [24]. According to this concept, health consists of multiple domains (e.g. bodily and mental functions, social and societal participation) and these domains were also recognized by workers with a low SEP [23].

Workers with a low SEP often face problems on multiple life domains [19], which could interfere with work functioning and health. In the interviews, workers with a low SEP recognized that not only health complaints are related to problems at work, but that problems in other life domains also interfere. Workers with a low SEP also mentioned that problems at work are often caused by underlying problems in other life domains that are not always identified by OHPs. OHPs and employers acknowledged in the focus groups that problems outside the workplace are relevant to discuss in occupational health practice and are often not identified. The time and energy that workers need for problems outside the workplace could negatively affect their work functioning [19]. Moreover, short term social or economic problems may hinder workers with a low SEP to improve their health on the longer term [19, 25]. For example, adherence to lifestyle interventions is often only feasible when short term problems in daily life are resolved [26, 27].

**Main determinants for health problems among workers with a low SEP**

Workers with a low SEP have a larger risk for health problems for three different reasons. First, unfavorable work-related determinants, including both physical and psychosocial factors. Physical factors prevalent among workers with a low SEP are biomechanical, chemical and biological exposures which increases the risk for physical health problems [6, 28, 29]. Workers with a low SEP also often have jobs that include repetitive work, heavy lifting and with poorer working arrangements, such as shift work [6, 30]. Psychosocial factors prevalent among workers with a low SEP are low job control, high job insecurity and low levels of social support [6, 28, 29, 31], which may result in a lower psychological wellbeing and an increased risk for mental health problems [32].

Second, unfavorable non-work-related determinants are more prevalent among workers with a low SEP. Workers with a low SEP more often have unhealthy lifestyle behaviors, such as smoking, physical inactivity, heavy drinking and unhealthy dietary patterns [19, 28, 33]. In addition, workers with a low SEP generally have limited financial resources, and these limited resources could hinder them to live healthy [25, 33]. Healthy behaviors are often more costly than unhealthy behaviors. For example, healthy food is often more expensive than unhealthy food [34]. Moreover, workers with a low SEP have more limited social networks than people with a higher SEP [23]. Social networks can provide resources, such as support or knowledge in enabling healthy behaviors [33]. Access to resources through social networks refers to the concept of ‘Social Capital’ [35]. Moreover, social capital may also be a work-related determinant, consisting of support from for example, the supervisor. People with a low SEP generally have lower levels of social capital which limits their access to obtain and use diverse resources [36]. This may lead to poorer health outcomes among people with a low SEP, as compared to people with a high SEP [35–37]. Hence, increasing social capital could be more important among workers with a low SEP than among workers with a high SEP, and the workplace could provide an opportunity to increase this.

Work and non-work-related determinants may also result in work-family conflicts, wherein family demands (i.e. non-work-related determinants) interfere with work life, and vice versa. Unfavorable work-related determinants such as shift work or less flexible work could negatively affect the family life [38]. Inversely, unfavorable non-work-related determinants, such as an unhealthy lifestyle could negatively affect the working life [39]. Work-family conflicts are associated with a higher sickness absence [40, 41] and poorer health outcomes [41, 42]. Especially among workers with a low SEP, work-family conflicts seem to have a more negative effect on health, compared to workers with a high SEP [43]. Hence, workers with a low SEP are simultaneously exposed to a variety of unfavorable determinants [6, 44]. Interventions that focus only on work-related determinants ignore the interconnections between these determinants and are less likely to be effective [44].

Third, poor health literacy to adapt these work and non-work-related determinants. Workers with a low SEP tend to have poor health literacy, which means that they have less cognitive and social skills which determine the motivation and ability of individuals to gain access to, understand and use information in ways that promote and maintain good health [33, 45]. As a result, workers with a low SEP may find it difficult to self-manage and adapt unfavorable circumstances in or outside the workplace, which could be caused by a lack of motivation or self-efficacy for their ability to adapt unfavorable circumstances [46]. Moreover, poor health literacy could also result from a lack of awareness and a lower risk perception of health problems. Workers with a low SEP hardly think about their own sustainable employability [19], which was also recognized in the interviews. Workers with a low SEP mentioned that it was difficult to be aware of a problem and to act on it. Especially when they were able to work they may not recognize the value or importance of changing unfavorable determinants for work functioning and health. Poor health literacy may lead to passive or avoidant coping styles towards health problems. Research shows that people with a higher SEP show a more active attitude towards their health status, whereas people with a low SEP focus more on acceptance instead of facing the challenges [22]. This could also be enhanced by the more difficult circumstances workers with a low SEP may face due to problems on multiple life domains. It may be harder for workers with a low SEP to act on these circumstances, making it easier to accept them. As a result, workers with a low SEP may be too late in addressing health problems, which could increase the risk for premature dropout from the labor market [47].

Only improving the ability of workers with a low SEP to self-manage and adapt health problems is not enough, this group of workers also need a supportive environment on how to perform the desired behavior. For example, a study among truck drivers showed that those who were motivated to change their lifestyle did not succeed, as they didn’t know how to overcome the obstacles in their work and private life [48]. For that reason, workers with a low SEP need support in tackling these problems, such as making an action plan, that includes information on how and when the behavior can be performed and thinking about strategies on how to overcome potential obstacles [49]. Moreover, workers with a low SEP also need a supportive environment as they have a lower control (i.e. autonomy) over decisions in and outside the workplace. Workers with a low SEP have, compared to workers with a high SEP, a lower decision latitude which is a predictor for health problems at the workplace [6]. Outside the workplace workers with a low SEP experience a lower control over decisions in their day-to-day lives, due to a lack of resources needed for health and wellbeing [25, 50]. Finally, supportive environments are associated with a decrease in work-family conflicts and an increase in social capital [35, 51, 52]. This could be relevant for workers with a low SEP, as they experience more negative health effects of work-family conflicts and have lower levels of social capital [36, 43]. So, to effectively self-manage and adapt problems on multiple life domains, relevant stakeholders (e.g. supervisor or partner) need to be involved in the decision-making process of solving problems. OHPs could play an important role in this process by bringing together the worker and relevant stakeholders.

A supportive environment can consist of an OHP who supports the worker in solving problems on multiple life domains. However, occupational health practice is mainly focused on healthy functioning at the workplace [53]. As a result, OHPs may insufficiently consider problems on other life domains than work or may lack competencies on how to support workers with a low SEP in solving problems on other life domains than work. Therefore, occupational health care should provide more attention to the interplay of problems in and outside the workplace and how this could affect work functioning and health of workers with a low SEP. Furthermore, preventive interventions wherein OHPs provide early support to workers with a low SEP could be difficult. OHPs are not always easily reached in organizations; they could be seen as someone who works for the employer (i.e. lack of trust) and workers could be unfamiliar with the preventive role of OHPs [54]. Finally, as was mentioned above, workers with a low SEP have a lower awareness and risk perception of health problems. As a result, workers with a low SEP do not easily ask for help from an OHP. For that reason, OHPs need to create a safe environment for workers with a low SEP and improve their familiarity among workers at the workplace.

#### Logic model of the problem

**Fig. 2:**
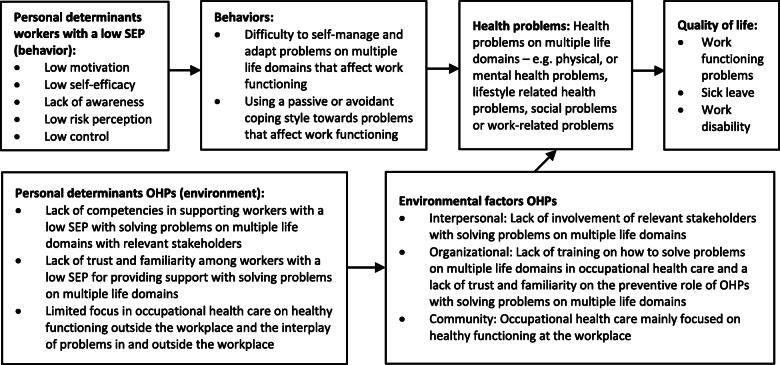
Logic model of the problem

To improve the health of workers with a low SEP from a broader perspective the PA should focus on identifying both work and non-work-related health problems, and also consider the interplay between these problems. Therefore, the program goal of the PA is to solve problems on multiple life domains that affect work functioning. This could result in healthy functioning at the workplace, sustainable employability and the prevention of work disability among workers with a low SEP. To achieve this, the logic model of the problem (Fig. 2) describes behavioral and environmental determinants that need to be considered in the PA. Behavioral determinants for workers with a low SEP are motivation, self-efficacy, awareness, risk perception and control for solving health problems on multiple life domains. Environmental determinants are competencies (knowledge and skills) for OHPs to support workers with a low SEP in solving health problems with relevant stakeholders, trust and familiarity of OHPs among workers with a low SEP and more attention for healthy functioning outside the workplace in occupational health care.

### Step 2: logic model of change

#### Performance objectives

The behavioral outcome related to goal of the PA is that workers with a low SEP are able to actively solve problems on multiple life domains that affect healthy functioning at work. The performance objectives associated with the behavioral outcome of workers with a low SEP are listed in Table 1. OHPs are the environmental agents at the workplace who can support workers with a low SEP. The environmental outcome related to the goal of the PA is that OHPs support workers with a low SEP in actively solving problems on multiple life domains that affect healthy functioning at work. The performance objectives associated with the environmental outcome are listed in Table 2. In additional file 2, the logic model of change can be found, which summarizes the behavioral and environmental determinants, performance objectives and outcomes.

**Table 1:** Performance objectives for the behavioral outcome

1. Identify problems in and/or outside the workplace that affect healthy functioning at work and select relevant stakeholders	
2. Actively prioritize problems in and/or outside the workplace that affect healthy functioning at work with relevant stakeholders	
3. Actively identify and find consensus on solutions for problems in and/or outside the workplace that affect healthy functioning at work with relevant stakeholders	
4. Implement solutions for problems in and/or outside the workplace that affect healthy functioning at work with relevant stakeholders

**Table 2:** Performance objectives for the environmental outcome

1. Discuss with the worker problems in and/or outside the workplace that affect healthy functioning at work and select relevant stakeholders	
2. Guide the worker and relevant stakeholder with actively prioritizing problems in and/or outside the workplace that affect healthy functioning at work	
3. Guide the worker and relevant stakeholder with actively identifying and finding consensus on solutions for problems in and/or outside the workplace that affect healthy functioning at work	
4. Support the worker with the implementation of solutions for problems in and/or outside the workplace that affect healthy functioning at work	

#### Selection of theoretical concepts and change objectives

To enable workers with a low SEP to actively identify, prioritize and solve problems in and/or outside the workplace the theoretical concepts of the Self-Determination Theory (SDT) were selected; autonomy, competence and relatedness. This theory argues that by increasing autonomy, competence and relatedness health related behaviors are more likely to be initiated and maintained (i.e. motivation) [55], and thereby may also positively influence the attitude of workers with a low SEP towards solving health problems (i.e. awareness and risk perception) [56]. The behavioral determinants control and self-efficacy described in the logic model of the problem match well with the determinants autonomy and competence. Furthermore, the key elements of the PA; involvement of relevant stakeholders and a consensus-based process match well with the determinants autonomy and relatedness. In additional file 3, matrices of change objectives can be found for the behavioral outcome to identify what workers with a low SEP may need to learn or change to achieve the performance objectives. For the environmental agents the theoretical concepts competence and attitude were selected. For OHPs to support workers with a low SEP, it is important that they feel competent, create a safe environment and have a positive attitude towards solving problems both in and outside the workplace. In additional file 4, matrices of change objectives can be found for the environmental outcome to identify what OHPs need to learn or change to achieve the performance objectives.

### Step 3: program design

Theory and evidence-based change methods and practical strategies were formulated in Tables 3 and 4 for the selected determinants of the behavioral and environmental outcome. The already existing protocol of the PA was used as a starting point for the delivery of practical strategies. This PA protocol exists of different steps that are considered logical and provide a structured way of understanding what problems and solutions are considered most relevant [58]. In applying the PA, a process leader is essential. OHPs are suitable for this role as they have communication skills, are independent, confidential and are used to guide workers with work-related problems. Furthermore, in the already existing protocol of the PA, the supervisor is often a relevant stakeholder for problems that are identified at the workplace [14]. The worker and supervisor can together decide on the most important problems and solutions, which will give a higher chance of solutions being implemented at the workplace. If problems are identified outside the workplace relevant stakeholders can vary, for example spouse, family members, friends or (health) professionals (e.g. general practitioner or job coach from the municipality). They can provide another perspective on the most important problems and solutions or can provide support in the implementation of solutions outside the workplace. The PA protocol, training and material need to be adapted to fit the goal of discussing and solving health problems both in and outside the workplace that may affect work functioning, and are presented in step 4: program production.

**Table 3:** Theoretical methods and practical strategies for selected determinants of the behavioral outcome

Determinant	Theory	Parameters	Practical strategies
Autonomy	Choice	Provide opportunities for choice	The worker and relevant stakeholder are both involved in the decision making of the most relevant problems and solutions in and/or outside the workplace
	Acknowledge feelings	Recognize perspectives of others	The OHP acknowledges the perspectives of the worker and relevant stakeholders on problems and solutions in and/or outside the workplace
	Personal responsibility	Identify values of behaviors and align with central values in life	Discuss consequences of problems and benefits of solving problems and choose solutions that could fit into the workplace and/or life outside the workplace
Competence	Social cognitive theory; self-efficacy [57]	Increase feelings of mastery	Find consensus on solutions, set specific solutions, break down solutions into smaller steps that are feasible to implement and compose action plans
		Involve relevant stakeholders	Problems and solutions are discussed with relevant stakeholders to assess different perspectives on the most relevant problems and solutions
		Provide feedback and evaluation	Find consensus on solutions and make an action plan that is feasible to implement and evaluate the implementation of the action plan
		Improve coping mechanisms	Reflect on potential barriers for the implementation of solutions and develop a plan to cope with these barriers
Relatedness	Social support	Support from OHP	The OHP provides tools to the worker to identify and prioritize problems and solutions in and/or outside the workplace
		Support from relevant stakeholders	Relevant stakeholders participate in the process of identifying and prioritizing problems in and/or outside the workplace and finding solutions
	Equality	Guidance by an independent person	OHP acknowledges all perspectives, remains impartial and generates consensus between the worker and the stakeholder
		A supportive environment to share problems and solutions	Being open and respectful to other perspectives on problems and solutions and OHP assures an equal involvement in the discussion
	Safety	A safe environment to share problems	OHP is confidential with the discussed problems and problems will only be discussed with other stakeholders if the worker agrees

**Table 4:** Theoretical methods and practical strategies for selected determinants of the environmental outcome

Determinant	Theory	Parameters	Practical strategies
Competence	Guided practice	Instruction and skills training	OHP receives a training on how to apply the PA with a broadened perspective and practice this in role plays
Attitude	Verbal persuasion	Providing arguments	Provide information on the Positive Health approach and why it is important to solve problems on multiple life domains with relevant stakeholders

### Step 4: program production

The existing protocol and material of the PA were adapted to match the broadened perspective of the PA (see Table 5). This resulted in an intervention that was named “Grip on Health”. The original PA materials were considered too complex (i.e. focus is put on the cognitive skills) for workers with a low SEP and too time consuming, also for the OHP [59]. As a result, there was a need to develop materials with more visual aspects that were less time consuming. In collaboration with a designer new material was developed that was tailored to the needs and wishes of workers with a low SEP and OHPs. The new material was pretested through interviews and focus groups among workers with a low SEP, OHPs and employers. Workers with a low SEP, as well as OHPs and employers, were positive towards the new material, considered the material useful to discuss problems in and outside the workplace and found that the material provided a structured way to identify problems and solutions. Pretesting the material also provided input for improvements in the material and practical requirements for working with the material in occupational health practice.

**Table 5:** The protocol of the Grip on Health intervention

Steps	Content
Step 1: Inventory	The process leader and worker discuss potential problems on multiple life domains
Step 2: Research	The process leader and worker prioritize problems that affect healthy functioning at work and discuss the causes and consequences of these problems
Step 3: Summary	The process leader and worker select the most relevant problems and decide which stakeholder is relevant to involve. The process leader invites the stakeholder and asks to think about problems for the worker
Step 4: Problem analysis	The process leader, worker and relevant stakeholder discuss the problems from their own perspective and reach consensus on the most relevant problems that affect healthy functioning at work
Step 5: Brainstorm	The process leader, worker and relevant stakeholder brainstorm about possible solutions
Step 6: Solution analysis	The process leader, worker and relevant stakeholder reach consensus on solutions
Step 7: Action plan	The process leader, worker and relevant stakeholder compose an action plan to implement solutions
Step 8: Evaluation	The process leader and worker evaluate the action plan. If necessary, an additional evaluation will be planned.

The training for OHPs was also adapted into a training for the Grip on Health intervention. The training will provide OHPs with information on 1) the variety of health problems among workers with a low SEP, 2) the Positive Health approach, 3) the PA and its key elements, 3) how to apply the Grip on Health intervention in practice, 4) how to act as a process leader and 5) how and when to involve relevant stakeholders in and outside the workplace. Information on the Grip on Health intervention will be alternated with role plays, giving OHPs the opportunity to practice certain steps of the intervention with the material and their role as process leader. The training will be given by two members of the planning group. At the end of the training, participating OHPs will receive a practical manual on how to apply the Grip on Health intervention, a presentation of the training and the materials of the intervention. Moreover, OHPs get a practical assignment, wherein they are asked to apply the intervention in occupational health practice among 3–5 workers with a low SEP. OHPs are advised to complete the steps of the intervention within 3 to 4 four different conversations within a time frame of 3 months. A couple of months after the training a follow up meeting will be planned in which OHPs will share their experiences with the practical assignment, reflect on the different steps of the intervention and on their role as process leader.

### Step 5: implementation plan

The experiences with the Grip on Health intervention in occupational health practice will be assessed in a pilot implementation study. We will invite approximately 20 OHPs for the Grip on Health training, and we will ask them to apply the intervention in their occupational health practice. Two important requirements were identified in the interviews and focus groups for optimal delivery of the intervention by the OHP. First, a confidential and safe environment are important preconditions for discussing problems at the workplace. OHPs that will be invited for the training need to have full confidentiality as problems from other life domains may also be discussed. In the Dutch context, OHPs need to be either physicians or nurses, or professionals who work under legal supervision of an occupational health physician. Furthermore, the OHP must also create a safe environment, as workers with a low SEP mentioned in the interviews that certain problems are difficult to discuss (e.g. problems outside the workplace) when they are not feeling safe. Second, the intervention cannot be applied in all situations or to all kinds of health problems. In the protocol of the PA it is stated that the PA is not suitable for a worker with a juridical conflict at work with for example the supervisor or for workers with serious medical conditions – e.g. severe mental disorders [14]. Moreover, OHPs and employers mentioned in the focus groups that not every non-work-related problem can be solved in the PA (e.g. financial problems) and that it may sometimes be better to refer a worker to a (health) professional from outside the workplace.

The trained OHPs will apply the intervention in an organization among workers with a low SEP. Therefore, the employers of the organization in which OHPs will apply the intervention are a relevant stakeholder for optimal delivery of the intervention. The employers need to allow and support the implementation of the Grip on Health intervention in their organization. As the needs assessment showed that workers with a low SEP do not easily ask for help from an OHP, employers and supervisors also need to make their workers with a low SEP aware of this intervention by referring a worker to an OHP when they notice health problems or problems that affect work functioning. Performance objectives for these environmental agents to enable implementation are listed in Table 6. To achieve these performance objectives, the OHP needs to provide employers with information and make them aware of the added value of the Grip on Health intervention. Employers will receive information from the planning group about the intervention and the OHP is asked to discuss with the employer how and when the intervention can be implemented.

**Table 6:** Performance objectives for employers

1. Employers are informed about the implementation of the Grip on Health intervention in their organization	
2. Employers are convinced of the added value of the Grip on Health intervention in their organization	
3. Employers approve that OHPs implement the Grip on Health intervention in their organization	
4. Employers facilitate time and sufficient resources for OHPs to implement the Grip on Health intervention in their organization	
5. Employers refer a worker to an OHP when they notice health problems or problems that affect work functioning.	

### Step 6: evaluation plan

To evaluate the pilot implementation of the Grip on Health intervention in occupational health practice we will use the Medical Research Council process evaluation framework [60]. In this framework the key components of a process evaluation are: measuring implementation (i.e. what is implemented and how?), mechanism of impact (how does the delivered intervention produce change?) and context (i.e. how does context affect implementation and outcomes). Implementation of interventions at the workplace may be difficult as it is dependent on how occupational health care is organized in an organization and on a variety of stakeholders, such as employers and supervisors. This in turn, emphasizes the need for conducting a more comprehensive process evaluation of the Grip on Health intervention with different methods (i.e. both qualitative and quantitative) and from different levels (i.e. workers with a low SEP, OHPs and other relevant stakeholders). The process of the implementation will be assessed by measuring the following aspects: 1) reach, 2) recruitment, 3) fidelity, 4) dose delivered, 5) dose received and 6) quality of delivery. The mechanisms of impact will be assessed by measuring 1) participant responsiveness (i.e. perceived satisfaction, effectiveness and relevance), and 2) perceived differentiation (i.e. essential components of the intervention). The context will be assessed by measuring the facilitators and barriers related to the implementation of the intervention in occupational health practice. First, a process evaluation will be conducted, because this information is essential to determine how, for whom and under what conditions the intervention will be feasible and applicable. Thereafter, we will use this information to decide whether and how we should conduct an effect-evaluation in occupational health practice. A randomized controlled trial is an appropriate method for an effect-evaluation [61], if this is considered feasible within occupational health practice [62].

## Discussion

This study describes how the PA was adapted to improve work functioning and health of workers with a low SEP from a broader perspective. Adaptation of the PA was guided by the IM protocol, which resulted in the Grip on Health intervention. In this intervention OHPs support workers with a low SEP in actively solving problems on multiple life domains that affect work functioning and thereby health. The intervention consists of a stepwise protocol to identify, prioritize and solve problems in and/or outside the workplace with the involvement of at least one relevant stakeholder. The OHP is considered the optimal professional to execute this intervention in daily practice as he or she already has an independent and confidential role in occupational health care.

Previous studies that used the IM protocol for the development of a PA intervention at the workplace focused on RTW [63, 64]. These studies based their intervention on the  Attitude Social influence Self-efficacy (ASE) model, as workers’ attitude, social influence and self-efficacy were identified as determinants for RTW. In this study the SDT was used as the needs assessment showed that workers with a low SEP may lack motivation to actively solve health problems, and according to this theory workers’ autonomy, competence and relatedness may increase their motivation for health-related behaviors [55]. This is important as workers with a low SEP use avoidant and/or passive coping styles towards health problems, which could increase the risk of further health deterioration and eventually the chance for premature dropout from the labor market. The concepts of the SDT, which are autonomy, competence and relatedness, are an essential part of the Grip on Health intervention and match well with the behavioral determinants self-efficacy and control that were described in the logic model of the problem. Moreover, participation of workers in the intervention could also increase the behavioral determinants awareness and risk perception towards health problems, which in turn may also improve the motivation of workers with a low SEP to solve these problems [65].

Implementation of the PA with a broadened perspective is beneficial for occupational health practice, as there is still too little awareness that aspects in multiple life domains may influence work functioning and it is therefore essential to take these into account to prevent work disability. This broadened perspective is also more in line with the Positive Health approach. In this approach, first a person evaluates each health domain for him or her selves, wherein the health status on each of these domains becomes visible. Then, a health professional asks the person what he or she wants to change to provide guidance in solving those problems that are really important to the person [24]. In that way, the Positive Health approach focuses on a person’s own responsibility, participation and self-management, which is also apparent in their definition of health: “Health as the ability to adapt and self-manage, in the light of physical, emotional and social challenges of life” [20]. However, one of the main points of criticism of the Positive Health approach is that not all people are equipped to manage problems themselves, especially people with a low SEP. For individuals with problems on multiple life domains an intervention wherein (health) professionals, social networks and organizations are involved is necessary to improve their health status [25]. The Grip on Health intervention tackles this point of criticism, as in the PA the OHP not only asks the worker what problems he or she wants to change but also involves relevant stakeholders and supports the worker in solving these problems.

### Methodological considerations

IM was a valuable tool to adapt the PA to the needs of the target group, workers with a low SEP. However, this is not a guarantee that the intervention will be successful. There are still some methodological considerations of the intervention itself. First, workers with a low SEP may be hard to reach for OHPs. The needs assessment showed that OHPs have a lack of trust and familiarity among workers with a low SEP. Therefore, OHPs are not easily approached or accessible as an health professional who can support them in solving health problems both in and outside the workplace. Furthermore, workers visit primarily a general practitioner when they are experiencing health problems outside the workplace. Integrating occupational and general health care might be a strategy to reach more workers in occupational health care [66]. For example, general practitioners could take into account work-related problems, be more aware of the importance of work as a contributory factor of health and if needed refer a worker to an OHP.

Second, it may also be challenging to involve relevant stakeholders from outside the workplace in an intervention that is facilitated and financed by the workplace. Stakeholders from outside the workplace could be the partner or family member of the worker, but also another health professional. However, including other health professionals for a face to face discussion with the worker and the OHP may too difficult to organize in practice, but will depend per situation. For example, in the Netherlands occupational health care is strictly separated from regular health care, which could make it harder to include health professionals from outside the workplace. In this study only stakeholders from the workplace were invited to participate in the focus groups, as their needs on how to adapt the PA were considered most relevant to consider for an intervention that will be implemented at the workplace. Nevertheless, adding views of professionals from outside the workplace on how to involve them in the intervention, could further improve the implementation of the intervention. Whether it is actually feasible in practice to involve stakeholders from outside the workplace needs to be further investigated.

Third, OHPs may also experience time as a barrier to implement the intervention in occupational health practice. Following the steps of the PA is a very time-consuming process [58, 67]. Nevertheless, the elaborated process of the PA gives OHPs the opportunity to get a complete overview of the worker and gain the workers’ trust in their guidance [58]. Gain the workers trust was mentioned as an important factor in this study for discussing health problems, especially for problems from outside the workplace. In this study different OHPs, which may vary in their possibilities to implement the Grip on Health intervention, will be trained to implement the intervention. Thereby, the pilot implementation study can provide more information on which type of OHPs would be most suitable for the implementation of this intervention, how much time is needed for the implementation of the intervention and whether implementation of this intervention is feasible.

## Conclusion

IM was a valuable tool for adaption of the PA to workers with a low SEP to improve their work functioning and health from a broader perspective. The IM provided information on which adaptations were needed to solve problems on multiple life domains that affect healthy functioning at work. This resulted in the Grip on Health intervention that is specifically tailored to workers with a low SEP and considers the interconnection between work and non-work-related determinants for work functioning and health. This intervention will be evaluated in a pilot implementation study to further explore whether and how this intervention fits in occupational health practice.

## Supplementary information

**Additional file 1.** Interview guides for the interviews and focus groups with workers with a low SEP, OHPs and employers.

**Additional file 2.** Logic model of chance. A summary of the behavioral and environmental determinants, performance objectives and outcomes.

**Additional file 3.** Matrices of change for the behavioral outcome. Change objectives for the behavioral outcome to identify what workers with a low SEP may need to learn or change to achieve the performance objectives.

**Additional file 4.** Matrices of change for the environmental outcome. Change objectives for the environmental outcome to identify what OHPs need to learn or change to achieve the performance objectives.

## Data Availability

The data generated and analyzed during the current study are not publicly available. The data consist of transcripts of interviews and focus groups which contain identifying information, and are therefore sensitive to privacy issues. The data are available from the corresponding author on reasonable request.

## Abbreviations

ASE: Attitude Social influence Self-efficacy
IM: Intervention Mapping
OHP: Occupational health professional
PA: Participatory Approach
RTW: Return to work
SDT: Self-Determination Theory
SEP: Socioeconomic Position
