# Supplementary material for: Improving the health of workers with a low socioeconomic position: Intervention Mapping as a useful method for adaptation of the Participatory Approach
Source: BMC Public Health. 2020 Jun 19;20:961. doi: 10.1186/s12889-020-09028-2 (PMC7304135; doi:10.1186/s12889-020-09028-2)
Supplement: Supplementary file 1 — Additional file 1. Interview guides for the interviews and focus groups with workers with a low SEP, OHPs and employers. [file 12889_2020_9028_MOESM1_ESM.docx]

**Additional file 1. Interview guides**

**Interview guide interviews**

1. What kind of (health) problems do you experience by yourself or among colleagues?
2. To what extent do you, or your colleagues, experience problems on multiple life domains?
3. What kind of life domains would you want to discuss at the workplace (e.g. work, lifestyle, etc.)?
4. With whom would you like to discuss problems on multiple life domains at the workplace?
5. In what kind of situation do you feel safe and confidential to discuss problems on multiple life domains?
6. In which way do you want to discuss problems on multiple life domains?
7. What do you think of the material for discussing problems?
8. What kind of information do you need for discussing solutions?
9. What do you think of the material for discussing solutions?
10. What do you think about making an action plan for solutions?
11. What kind of aspects do you want in an action plan?
12. What do you think of the material for making an action plan?
13. What do you think about the evaluation of an action plan?
14. What kind of aspects do you want to be in an evaluation?
15. What do you think of the material for an evaluation?
16. What do you think of inviting other people (e.g. supervisor, partner) to the conversations?
17. In which way could workers be reached for a preventive intervention?

**Interview guide focus groups**

1. What kind of life domains could be discussed with workers at the workplace (e.g. work, lifestyle, etc.)?
2. What kind of problems on multiple life domains could be discussed with workers at the workplace?
3. Which professionals at the workplace could implement this intervention?
4. In what way would workers want to discuss problems on multiple life domains?
5. What do you think of the material for discussing problems on multiple life domains?
6. What kind of information do you need for discussing solutions?
7. What do you think of the material for discussing solutions?
8. What kind of solutions can be offered to workers?
9. What kind of information do you need for making an action plan?
10. What do you think of the material for making an action plan?
11. What kind of information do you need for the evaluation?
12. What do you think of the material for the evaluation?
13. To what extent is it needed to invite other people (e.g. supervisor, partner) to the conversations?
14. To what extent is it feasible to invite other people (e.g. supervisor, partner), to the conversations?
15. In which way could employees be reached for this preventive intervention?
16. What is the added value of this intervention?
