# Supplementary material for: Improving the health of workers with a low socioeconomic position: Intervention Mapping as a useful method for adaptation of the Participatory Approach
Source: BMC Public Health. 2020 Jun 19;20:961. doi: 10.1186/s12889-020-09028-2 (PMC7304135; doi:10.1186/s12889-020-09028-2)
Supplement: Supplementary file 2 — Additional file 2. Logic model of chance. A summary of the behavioral and environmental determinants, performance objectives and outcomes. [file 12889_2020_9028_MOESM2_ESM.docx]

**Additional file 2. Logic model of change**

**Performance objectives (behavior)**

1. Identify problems in and/or outside the workplace that affect healthy functioning at work and select relevant stakeholders
2. Actively prioritize problems in and/or outside the workplace that affect healthy functioning at work with relevant stakeholders
3. Actively identify and find consensus on solutions for problems in and/or outside the workplace that affect healthy functioning at work with relevant stakeholders
4. Implement solutions for problems in and/or outside the workplace that affect healthy functioning at work with relevant stakeholders

**Personal determinants workers with a low SEP (behavior)**

- Motivation: be motivated to solve problems
- Self-efficacy: feel confident to solve problems
- Awareness: be aware of problems
- Risk perception: Value the importance of solving problems
- Control: be able to make decisions on how to solve problems

**Behavioral outcome:**

Actively solving problems on multiple life domains that affect healthy functioning at work

**Health and quality of life:**

- Healthy functioning at work
- Sustainable employability
- Prevention of work disability

**Personal determinants OHPs and relevant stakeholders (environment):**

- Competencies for supporting workers with a low SEP in solving problems on multiple life domains
- Being trusted and being familiar among workers with a low SEP for support in solving problems on multiple life domains
- Value the importance of solving problems on multiple life domains

**Performance objectives (environment)**

1. Discuss with the worker problems in and/or outside the workplace that affect healthy functioning at work and select relevant stakeholders
2. Guide the worker and relevant stakeholders with actively prioritizing problems in and/or outside the workplace that affect healthy functioning at work
3. Guide the worker and relevant stakeholder with actively identifying and finding consensus on solutions for problems in and/or outside the workplace that affect healthy functioning at work
4. Support the worker with the implementation of solutions for problems in and/or outside the workplace that affect healthy functioning at work

**Environmental outcome (organizational level):**

OHP supports the worker with a low SEP in actively solving problems on multiple life domains that affect healthy functioning at work
