# Supplementary material for: Improving the health of workers with a low socioeconomic position: Intervention Mapping as a useful method for adaptation of the Participatory Approach
Source: BMC Public Health. 2020 Jun 19;20:961. doi: 10.1186/s12889-020-09028-2 (PMC7304135; doi:10.1186/s12889-020-09028-2)
Supplement: Supplementary file 3 — Additional file 3. Matrices of change for the behavioral outcome. Change objectives for the behavioral outcome to identify what workers with a low SEP may need to learn or change to achieve the performance objectives. [file 12889_2020_9028_MOESM3_ESM.docx]

**Additional file 3. Matrices of change for the behavioral outcome**

| **Behavioral outcome: Actively solving problems on multiple life domains that affect healthy functioning at work** | | | |
| --- | --- | --- | --- |
| **Performance objectives** | **Autonomy** | **Competence** | **Relatedness** |
| 1: Identify problems in and/or outside the workplace that affect healthy functioning at work and select relevant stakeholders | Being able to discuss problems in and/or outside the workplace with an OHP and to choose which stakeholder to involve | Feel confident in the ability to identify problems in and/or outside the workplace with an OHP | Experience a safe environment to discuss problems in and/or outside the workplace with an OHP |
| 2: Actively prioritize problems in and/or outside the workplace that affect healthy functioning at work with relevant stakeholders | Being able to prioritize most relevant problems in and/or outside the workplace or understand and accept the perspective of the stakeholder on problems | Feel confident in the ability to prioritize the most relevant problems in and/or outside the workplace with the stakeholder | Experience a safe environment and feel support from the OHP and the stakeholder to prioritize the most relevant problems in and/or outside the workplace |
| 3: Actively identify and find consensus on solutions for problems in and/or outside the workplace that affect healthy functioning at work with relevant stakeholders | Being able to choose solutions for problems in and/or outside the workplace or to understand and accept the perspective of the stakeholder on solutions | Feel confident in the ability to find solutions for problems in and/or outside the workplace with the stakeholder | Experience a safe environment and feel support from the OHP and the stakeholder in finding solutions for problems in and/or outside the workplace |
| 4: Implement solutions for problems in and/or outside the workplace that affect healthy functioning at work with relevant stakeholders | Being able to implement solutions for problems in and/or outside the workplace or be involved in implementation of solutions by the stakeholder | Express confidence in the ability to implement solutions or that the stakeholder implements solutions for problems in and/or outside the workplace | Experience a safe environment and feel support from the OHP and the stakeholder for implementation of solutions for problems in and/or outside the workplace |
