# Supplementary material for: Improving the health of workers with a low socioeconomic position: Intervention Mapping as a useful method for adaptation of the Participatory Approach
Source: BMC Public Health. 2020 Jun 19;20:961. doi: 10.1186/s12889-020-09028-2 (PMC7304135; doi:10.1186/s12889-020-09028-2)
Supplement: Supplementary file 4 — Additional file 4. Matrices of change for the environmental outcome. Change objectives for the environmental outcome to identify what OHPs need to learn or change to achieve the performance objectives. [file 12889_2020_9028_MOESM4_ESM.docx]

**Additional file 4. Matrices of change for the environmental outcome**

| **Environmental outcome: Support workers with a low SEP in solving problems on multiple life domains that affect healthy functioning at work** | | |
| --- | --- | --- |
| **Performance objectives** | **Competence** | **Attitude** |
| 1: Discuss with the worker problems in and/or outside the workplace that affect healthy functioning at work and select relevant stakeholders | Being able to discuss problems in and/or outside the workplace with workers with a low SEP and to invite relevant stakeholders | Being positive towards identifying problems in and/or outside the workplace with workers with a low SEP and create a safe environment for the worker |
| 2: Guide the worker and relevant stakeholder with actively prioritizing problems in and/or outside the workplace that affect healthy functioning at work | Being able to guide the worker and stakeholder with identifying the most relevant problems in and/or outside the workplace and involve the perspectives of the worker and the stakeholder on problems | Create a safe and supportive environment for the worker and the stakeholder to share perspectives on problems, remain impartial and only give advice on the most relevant problems |
| 3: Guide the worker and relevant stakeholder with actively identifying and finding consensus on solutions for problems in and/or outside the workplace that affect healthy functioning at work | Being able to guide the worker and the stakeholder with identifying the most relevant solutions for problems in and/or outside the workplace and to involve the perspective of both the worker and the stakeholder on solutions | Create a safe and supportive environment for the worker and the stakeholder to share perspectives on solutions, remain impartial and only give advice on the most relevant solutions |
| 4: Support the worker with the implementation of solutions for problems in and/or outside the workplace that affect healthy functioning at work | Being able to coach workers with a low SEP on how to implement solutions for problems in and/or outside the workplace or on how the stakeholder can implement solutions for the worker | Create a supportive environment for the worker in the implementation of solutions and coach the worker on the implementation of solutions |
